# Coefficient of variation of platelet count as a prognostic Indicator for intracerebral hemorrhage: findings from a multicenter cohort

**DOI:** 10.3389/fneur.2026.1868807

**Published:** 2026-08-19

**Authors:** Tao Ge, Mengmeng Qiu, Taotao Shi, Dachang Qiu, Hao Wang, Pandi Chen, Maosong Chen

**Affiliations:** 1 Department of Neurosurgery, The Affiliated Lihuili Hospital of Ningbo University, Ningbo, Zhejiang, China; 2 The Affiliated Hospital of Qingdao University, Qingdao Medical College, Qingdao University, Qingdao, Shandong, China

**Keywords:** coefficient of variation, external validation, intracerebral hemorrhage, MIMIC-IV database, platelet count, prognostic indicator

## Abstract

**Background:**

Platelet count fluctuations are common in patients with intracerebral hemorrhage (ICH), but the prognostic relevance of these fluctuations remains uncertain.

**Methods:**

We conducted a retrospective multicenter cohort study using MIMIC-IV as the primary cohort and an independent Chinese cohort for external validation. Platelet count CV was calculated as the sample standard deviation divided by the mean of at least three observed, nonmissing platelet measurements obtained during the index hospitalization within 15 days before ICU admission. Missing measurement fields were excluded and were not treated as zero. Logistic regression, Cox models, restricted cubic splines, Kaplan–Meier analyses, subgroup analyses, reclassification metrics, and sensitivity analyses were performed. Primary multivariable models additionally adjusted for platelet measurement count, and effect estimates were reported per 0.1-unit increase in CV.

**Results:**

The primary cohort included 2,202 patients, of whom 294 died during the index hospitalization. After adjustment for clinical covariates and platelet measurement count, each 0.1-unit increase in platelet count CV was associated with in-hospital mortality (OR, 1.21; 95% CI, 1.07–1.38; *p* = 0.003), 28-day mortality (HR, 1.14; 95% CI, 1.04–1.26; *p* = 0.006), 90-day mortality (HR, 1.11; 95% CI, 1.02–1.21; *p* = 0.014), and 360-day mortality (HR, 1.11; 95% CI, 1.02–1.20; *p* = 0.016). Results were consistent in fixed-three-measurement and landmark sensitivity analyses. Adding platelet count CV produced modest reclassification improvement (continuous NRI, 0.209; 95% CI, 0.079–0.372; IDI, 0.007; 95% CI, 0.001–0.019). In the external cohort, platelet count CV was associated with 28-day mortality after multivariable adjustment (HR per 0.1-unit increase, 1.141; 95% CI, 1.007–1.292; *p* = 0.042).

**Conclusion:**

Higher pre-ICU platelet count CV was associated with mortality after adjustment for clinical covariates and platelet-monitoring frequency. The association was directionally reproduced in the external cohort, although the effect estimate was modest. Prospective studies with standardized platelet-sampling schedules are needed before clinical implementation.

## 1 Introduction

Intracerebral hemorrhage (ICH) carries high mortality and disability, yet its complex pathophysiological mechanisms remain incompletely understood (1–3). Platelets are a critical cellular component of hemostasis, and thrombocytopenia is a common hematological abnormality in neurocritical care (4–8). Previous studies have associated lower platelet counts with adverse outcomes after ICH (6, 9–11), whereas the clinical benefit of acute platelet-modifying strategies, particularly platelet transfusion, remains uncertain (12). However, the prognostic relevance of fluctuations in platelet count over time has not been systematically characterized in patients with ICH.

We therefore used the coefficient of variation (CV) to quantify temporal variability in platelet count. Using a large US critical-care database and an independent Chinese hospital cohort, we evaluated the association between pre-ICU platelet count CV and mortality, examined the influence of platelet-monitoring frequency, and assessed the incremental and externally validated prognostic information provided by this marker.

## 2 Methods

### 2.1 Data sources and ethical approval

Data for the primary cohort were extracted from the MIMIC-IV database. This collaborative repository was jointly developed by the Massachusetts Institute of Technology (MIT) and Beth Israel Deaconess Medical Center (BIDMC), containing de-identified electronic health records (EHRs) from intensive care unit (ICU) and inpatient admissions at BIDMC spanning the 2008–2022 period (version 3.1). The database initiative received approval from the BIDMC Institutional Review Board (IRB), which waived formal ethical review for all secondary research utilizing this resource, contingent upon investigators completing mandatory training and certification (13). Author QIU successfully fulfilled the required training and examination requirements, and was granted authorized access to the MIMIC-IV database (CITI Program Record ID: 65770488).

### 2.2 External validation cohort

The Chinese cohort enrolled from the Affiliated Lihuili Hospital of Ningbo University, was set as the independent external validation cohort in this study. This study was reviewed and approved by the Medical Ethics Committee of the Affiliated Lihuili Hospital of Ningbo University (approval number: KY2026SL163–01). Given the retrospective study design and complete de-identification of all patient records, the Medical Ethics Committee waived the requirement for written informed consent from study participants.

### 2.3 Data extraction

Data extraction was performed using Structured Query Language. Patients were identified using International Classification of Diseases, Ninth and Tenth Revision codes for non-traumatic ICH (ICD-9 code 431 and ICD-10 codes I61.0-I61.7 and I61.9). Demographic variables, physiological measurements, laboratory data, treatments, comorbidities, ICH location, severity scores, and outcomes were extracted. Eligible platelet measurements were linked by subject and hospital-admission identifiers so that measurements were restricted to the same index hospitalization.

### 2.4 Study population and eligibility criteria

We identified hospitalized patients with confirmed non-traumatic ICH in MIMIC-IV and the Chinese external validation cohort who were admitted to an ICU during the index hospitalization. For patients with multiple ICU admissions, only the first ICU admission was included. ICU admission was defined as time zero for outcome follow-up. The primary exposure window extended from the later of hospital admission or 15 days before ICU admission to immediately before ICU admission; measurements from previous hospitalizations and measurements obtained after ICU admission were excluded from the primary exposure. Exact ICH symptom-onset time was not reliably available in MIMIC-IV; therefore, the exposure window represented pre-ICU in-hospital platelet variability rather than an onset-based window.

**Figure 1:**
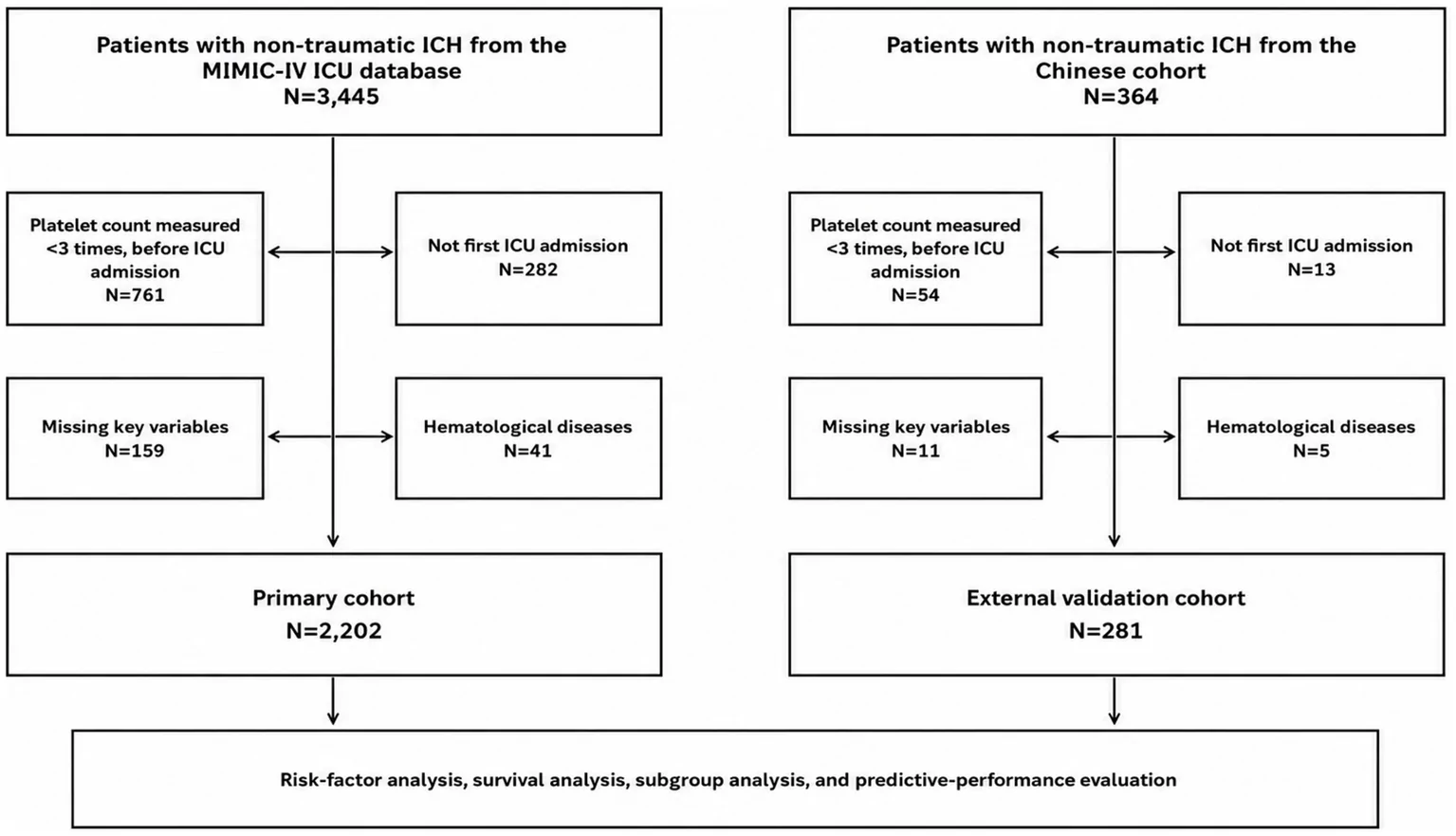
Flowchart of patient inclusion and exclusion in the primary and external validation cohorts.

Patients were required to have at least three observed, nonmissing platelet count measurements within the predefined pre-ICU window. Exclusion criteria were: (1) a non-index ICU admission; (2) a hematological disease that could directly affect platelet count or variability; (3) missing key study variables; or (4) fewer than three observed platelet measurements in the eligible window. The cohort-selection process is shown in Figure 1.

### 2.5 Definition of platelet count variability

For each patient, platelet count CV was calculated using all observed platelet count measurements in the eligible pre-ICU window. Empty or unavailable measurement fields were treated as missing and were not assigned a value of zero. CV was defined as the sample standard deviation divided by the arithmetic mean of the observed platelet counts.

Platelet count CV = sample standard deviation of platelet count/mean platelet count.

Platelet count CV was unitless. In all regression analyses, CV was modeled continuously and ORs and HRs were reported per 0.1-unit absolute increase. Only pre-ICU measurements were used in the primary exposure definition; mortality follow-up began at ICU admission.

### 2.6 Definition of outcome variables

The primary outcome was all-cause mortality during the index hospitalization after ICU admission. Secondary time-to-event outcomes were 28-day, 90-day, and 360-day all-cause mortality after ICU admission. Patients who remained alive at the end of the corresponding follow-up horizon were censored.

### 2.7 Sensitivity analyses

To address monitoring-frequency bias, we compared patients included with at least three observed pre-ICU measurements with patients who had only one or two measurements, using standardized mean differences. We also refitted the primary logistic and Cox models with additional adjustment for the total number of eligible platelet measurements. In a fixed-measurement sensitivity analysis, platelet count CV was recalculated using only the last three nonmissing pre-ICU platelet measurements for every patient, and the models were refitted with and without adjustment for total measurement count. Exact sampling timestamps were unavailable; therefore, the interval between the first and last eligible measurements could not be included as an additional adjustment variable.

We additionally performed 3-day and 7-day ICU-period landmark analyses. For each landmark, platelet count CV was recalculated using measurements obtained within the corresponding period after ICU admission; patients who died before the landmark or had fewer than three measurements within the landmark window were excluded, and follow-up began at the landmark. To explore confounding by ICH-specific imaging severity, separate external-cohort sensitivity models were fitted that included hematoma volume and intraventricular hemorrhage (IVH), together with the clinical covariates specified in Section 2.8. Complete admission CT imaging data were available for all 281 patients in the external validation cohort. Hematoma volume was estimated from the admission CT reports, which were interpreted by experienced senior clinical radiologists.

### 2.8 Statistical analysis

Data cleaning and statistical analyses were performed using R software, version 4.4.1. Continuous variables were summarized as the mean with standard deviation when normally distributed and as the median with interquartile range when non-normally distributed. Categorical variables were presented as frequencies and percentages. Between-group comparisons used Student’s t test or the Mann–Whitney U test for continuous variables and the chi-square test or Fisher’s exact test for categorical variables, as appropriate. Variables with missingness greater than 35% were excluded. Variables with more than 35% missingness were excluded, and multiple imputation was used for variables with lower levels of missingness.

Univariable logistic regression was used to evaluate associations with in-hospital mortality. Clinically relevant covariates and variables identified in the univariable assessment were included in multivariable logistic regression. Because laboratory-monitoring intensity was a central potential source of bias, the primary multivariable model additionally included platelet measurement count. Cox proportional hazards models were used for 28-day, 90-day, and 360-day mortality and likewise included platelet measurement count. ORs and HRs for platelet count CV were reported per 0.1-unit increase.

Restricted cubic spline analyses were used to assess nonlinearity and were adjusted using the same covariates as the corresponding primary model, including platelet measurement count. Kaplan–Meier curves were generated using the cohort-specific median CV and Youden-index-derived cutoff, and survival distributions were compared by log-rank tests. Prespecified subgroup Cox models were adjusted for platelet measurement count, age, sex, and SOFA score; the subgroup-defining variable was omitted from its corresponding model, and interaction terms were used to evaluate effect modification.

The incremental prognostic value of platelet count CV was evaluated using continuous net reclassification improvement (NRI) and integrated discrimination improvement (IDI). The baseline model included GCS, SOFA, APS III, and platelet measurement count, and the extended model additionally included platelet count CV. Standardized mean differences were used to compare included and excluded patients, with an absolute SMD of at least 0.10 considered potentially meaningful.

**Figure 2:**
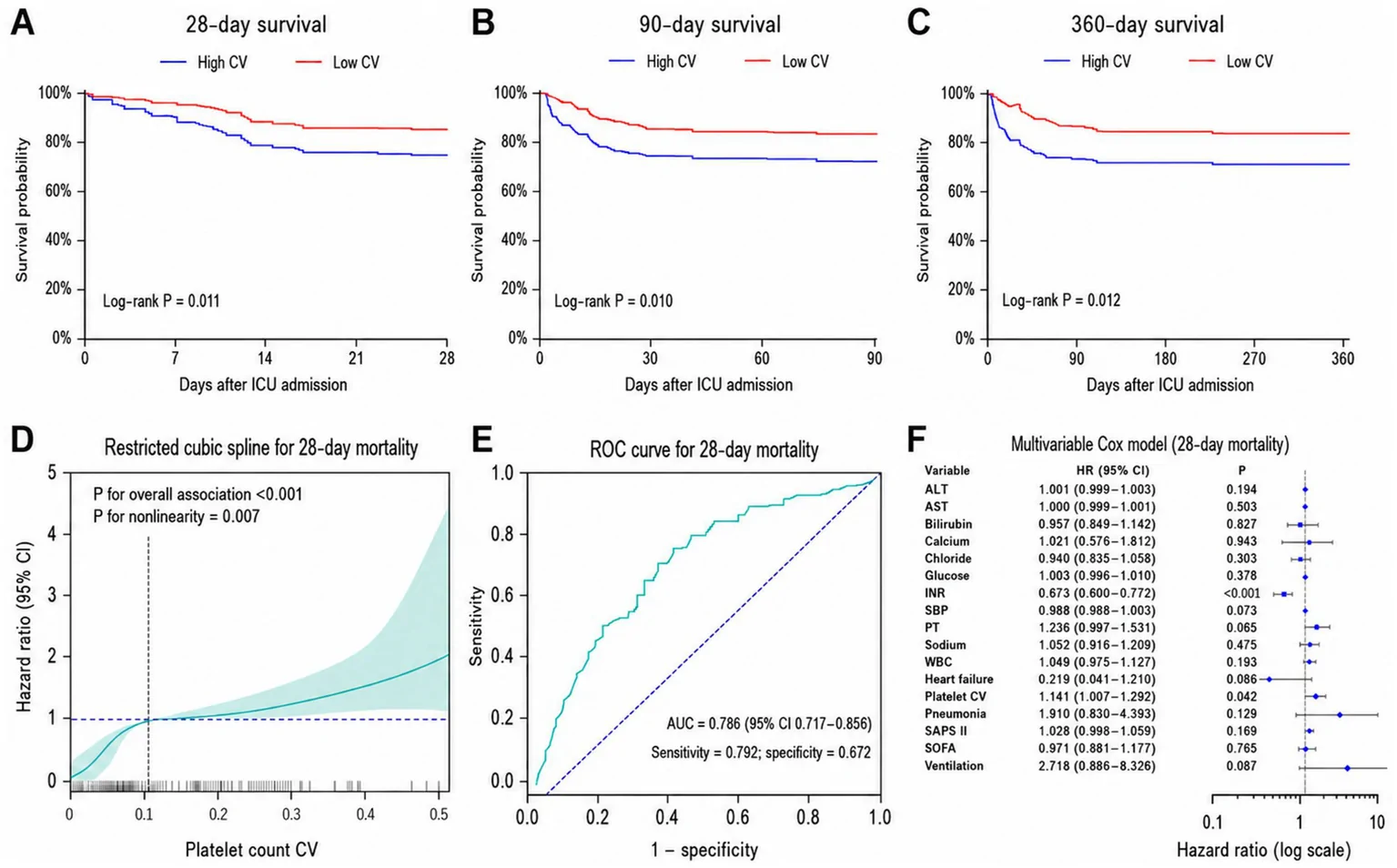
**(A–C)** Kaplan–Meier survival curves for 28-day, 90-day, and 360-day mortality in the external validation cohort, stratified using the Youden-index-derived platelet count CV cutoff. **(D)** Restricted cubic spline analysis of platelet count CV and 28-day mortality. **(E)** Receiver operating characteristic curve for 28-day mortality; the AUC was 0.786 (95% CI, 0.717–0.856), with sensitivity of 0.792 and specificity of 0.672. **(F)** Forest plot of the multivariable Cox proportional hazards model for 28-day mortality. The HR for platelet count CV is reported per 0.1-unit increase. AUC, area under the curve; CI, confidence interval; CV, coefficient of variation; HR, hazard ratio.

In the external validation cohort, receiver operating characteristic analysis was used to assess discrimination for 28-day mortality. The multivariable Cox model displayed in Figure 2F included ALT, AST, total bilirubin, calcium, chloride, glucose, INR, systolic blood pressure, prothrombin time, sodium, white blood cell count, heart failure, pneumonia, SAPS II, SOFA, mechanical ventilation, and platelet count CV. Separate imaging-adjusted sensitivity models included age, sex, GCS, SOFA, baseline platelet count, platelet measurement count, acute kidney injury, pneumonia, chronic kidney disease, myocardial infarction, anticoagulant use, surgery, CRRT, mechanical ventilation, platelet transfusion, ICH location, race, hematoma volume, and IVH. All 281 patients had complete imaging information from admission CT examinations. Hematoma volume was estimated from the corresponding radiology reports interpreted by experienced senior clinical radiologists. All tests were two-sided and *p* < 0.05 was considered statistically significant.

## 3 Results

### 3.1 Baseline characteristics

Among 3,445 patients with non-traumatic ICH initially identified in MIMIC-IV, 761 had fewer than three observed pre-ICU platelet measurements, 282 were not undergoing their first ICU admission, 159 had missing key variables, and 41 had hematological diseases affecting platelet counts. The primary cohort therefore included 2,202 patients (Figure 1), of whom 294 died during the index hospitalization and 1,908 survived, corresponding to an in-hospital mortality rate of 13.4%.

The median age was 70.0 years (IQR, 58.0–80.0), and 54.3% of patients were male. Non-survivors had higher SOFA, APS III, and SAPS II scores and higher frequencies of acute kidney injury, pneumonia, myocardial infarction, surgery, continuous renal replacement therapy, and mechanical ventilation. The median platelet count CV was 0.13 (IQR, 0.08–0.22) overall, 0.13 (IQR, 0.08–0.21) among survivors, and 0.17 (IQR, 0.11–0.27) among non-survivors (*p* < 0.001). The median number of eligible platelet measurements was 8 (IQR, 5–13) overall, 8 (IQR, 5–14) among survivors, and 7 (IQR, 4–13) among non-survivors (*p* = 0.045; Table 1).

**Table 1:** Baseline characteristics of the primary cohort.

Variable	Overall	Survivors	Non-survivors	*p*-value
Age, years	70.00 (58.00–80.00)	69.00 (58.00–80.00)	72.50 (60.00–82.00)	0.012
Weight, kg	76.20 (64.22–90.29)	76.20 (64.28–90.60)	76.85 (64.33–89.85)	0.712
GCS	14.00 (11.00–15.00)	14.00 (12.00–15.00)	15.00 (10.00–15.00)	0.063
SOFA	3.00 (2.00–5.00)	3.00 (2.00–4.00)	4.00 (3.00–7.00)	<0.001
APS III	35.00 (26.00–45.00)	34.00 (26.00–43.00)	44.00 (32.25–59.00)	<0.001
SAPS II	32.00 (25.25–40.00)	31.00 (25.00–38.00)	38.00 (32.00–47.00)	<0.001
Heart rate, bpm	81.00 (71.00–93.00)	81.00 (71.00–92.00)	82.00 (71.00–96.00)	0.115
Respiratory rate, breaths/min	18.00 (15.00–21.00)	18.00 (15.00–21.00)	18.00 (15.00–22.00)	0.493
NBPS, mmHg	135.00 (120.00–150.00)	135.00 (121.00–150.00)	132.50 (116.00–150.00)	0.068
NBPD, mmHg	74.00 (64.00–87.00)	75.00 (64.00–87.00)	70.50 (60.00–83.00)	<0.001
Temperature, °F	98.30 (97.80–98.80)	98.30 (97.80–98.80)	98.25 (97.70–98.90)	0.449
SpO2, %	98.00 (96.00–100.00)	98.00 (96.00–100.00)	99.00 (96.00–100.00)	<0.001
Hematocrit, %	37.05 (32.90–40.40)	37.20 (33.10–40.50)	35.25 (31.30–39.50)	<0.001
Hemoglobin, g/dL	12.20 (10.80–13.50)	12.30 (10.90–13.60)	11.75 (10.10–13.10)	<0.001
RDW, %	13.80 (13.10–14.80)	13.70 (13.00–14.70)	14.10 (13.40–15.20)	<0.001
RBC, M/μL	4.08 (3.60–4.50)	4.10 (3.63–4.52)	3.91 (3.38–4.36)	<0.001
WBC, K/μL	10.00 (7.90–12.88)	9.90 (7.80–12.50)	11.70 (8.90–15.07)	<0.001
Platelet count CV	0.13 (0.08–0.22)	0.13 (0.08–0.21)	0.17 (0.11–0.27)	<0.001
Platelet count, K/μL	209.00 (165.00–259.00)	211.50 (168.00–260.12)	189.50 (142.12–241.25)	<0.001
Platelet count measurements	8.00 (5.00–13.00)	8.00 (5.00–14.00)	7.00 (4.00–13.00)	0.045
Anion gap, mmol/L	14.00 (12.00–16.00)	14.00 (12.00–16.00)	15.00 (12.25–17.00)	0.002
Calcium, mg/dL	8.80 (8.40–9.20)	8.80 (8.40–9.20)	8.60 (8.10–9.00)	<0.001
Chloride, mEq/L	104.00 (101.00–107.00)	103.50 (101.00–106.00)	104.50 (101.00–108.00)	0.007
Glucose, mg/dL	127.00 (107.00–155.75)	125.00 (105.75–151.00)	145.50 (119.00–189.50)	<0.001
Potassium, mEq/L	3.90 (3.60–4.30)	3.90 (3.60–4.30)	4.00 (3.70–4.40)	<0.001
Sodium, mEq/L	139.00 (137.00–142.00)	139.00 (137.00–142.00)	140.00 (137.00–142.00)	0.251
INR	1.10 (1.10–1.30)	1.10 (1.10–1.20)	1.20 (1.10–1.30)	<0.001
PT, s	12.60 (11.70–13.90)	12.50 (11.60–13.80)	13.00 (12.10–14.78)	<0.001
PTT, s	28.30 (25.80–31.20)	28.30 (25.80–31.02)	28.70 (25.30–32.08)	0.243
ALT, U/L	21.00 (14.00–33.00)	21.00 (14.00–33.00)	23.00 (16.00–37.50)	0.015
AST, U/L	27.00 (19.00–41.75)	26.00 (19.00–40.00)	33.00 (23.00–56.00)	<0.001
Total bilirubin, mg/dL	0.60 (0.40–0.80)	0.60 (0.40–0.80)	0.65 (0.40–1.00)	0.003
Creatinine, mg/dL	0.90 (0.70–1.10)	0.90 (0.70–1.10)	1.00 (0.80–1.50)	<0.001
Urea nitrogen, mg/dL	16.00 (12.00–22.00)	16.00 (12.00–21.00)	19.00 (14.00–29.75)	<0.001
Race				<0.001
White	1,316 (59.8%)	1,185 (62.1%)	131 (44.6%)	
Black	220 (10.0%)	194 (10.2%)	26 (8.8%)	
Asian	90 (4.1%)	79 (4.1%)	11 (3.7%)	
Others	576 (26.2%)	450 (23.6%)	126 (42.9%)	
Sex				0.664
Female	1,007 (45.7%)	876 (45.9%)	131 (44.6%)	
Male	1,195 (54.3%)	1,032 (54.1%)	163 (55.4%)	
Platelet transfusion				<0.001
No	2083 (94.6%)	1822 (95.5%)	261 (88.8%)	
Yes	119 (5.4%)	86 (4.5%)	33 (11.2%)	
ICH location				0.011
Deep hemorrhage (basal ganglia/internal capsule)	169 (7.7%)	151 (7.9%)	18 (6.1%)	
Cerebral lobe	386 (17.5%)	343 (18.0%)	43 (14.6%)	
Brainstem	43 (2.0%)	32 (1.7%)	11 (3.7%)	
Cerebellum	110 (5.0%)	96 (5.0%)	14 (4.8%)	
Ventricle	257 (11.7%)	208 (10.9%)	49 (16.7%)	
Multifocal hemorrhage	21 (1.0%)	17 (0.9%)	4 (1.4%)	
Others	1,216 (55.2%)	1,061 (55.6%)	155 (52.7%)	
AKI				<0.001
No	1763 (80.1%)	1,580 (82.8%)	183 (62.2%)	
Yes	439 (19.9%)	328 (17.2%)	111 (37.8%)	
Hypertension				0.202
No	847 (38.5%)	724 (37.9%)	123 (41.8%)	
Yes	1,355 (61.5%)	1,184 (62.1%)	171 (58.2%)	
Liver cirrhosis				<0.001
No	2,127 (96.6%)	1856 (97.3%)	271 (92.2%)	
Yes	75 (3.4%)	52 (2.7%)	23 (7.8%)	
Hepatitis				0.807
No	2,161 (98.1%)	1873 (98.2%)	288 (98.0%)	
Yes	41 (1.9%)	35 (1.8%)	6 (2.0%)	
Pneumonia				<0.001
No	1,689 (76.7%)	1,507 (79.0%)	182 (61.9%)	
Yes	513 (23.3%)	401 (21.0%)	112 (38.1%)	
CKD				0.014
No	1925 (87.4%)	1,681 (88.1%)	244 (83.0%)	
Yes	277 (12.6%)	227 (11.9%)	50 (17.0%)	
Cancer				0.100
No	1853 (84.2%)	1,596 (83.6%)	257 (87.4%)	
Yes	349 (15.8%)	312 (16.4%)	37 (12.6%)	
Type 2 diabetes				0.007
No	1,660 (75.4%)	1,457 (76.4%)	203 (69.0%)	
Yes	542 (24.6%)	451 (23.6%)	91 (31.0%)	
Hyperlipidemia				0.264
No	1,305 (59.3%)	1,122 (58.8%)	183 (62.2%)	
Yes	897 (40.7%)	786 (41.2%)	111 (37.8%)	
Heart failure				0.007
No	1881 (85.4%)	1,645 (86.2%)	236 (80.3%)	
Yes	321 (14.6%)	263 (13.8%)	58 (19.7%)	
Myocardial infarction				<0.001
No	2,114 (96.0%)	1845 (96.7%)	269 (91.5%)	
Yes	88 (4.0%)	63 (3.3%)	25 (8.5%)	
COPD				0.258
No	2015 (91.5%)	1751 (91.8%)	264 (89.8%)	
Yes	187 (8.5%)	157 (8.2%)	30 (10.2%)	
Anticoagulant use				0.034
No	2011 (91.3%)	1733 (90.8%)	278 (94.6%)	
Yes	191 (8.7%)	175 (9.2%)	16 (5.4%)	
Antiplatelet use				0.801
No	2023 (91.9%)	1754 (91.9%)	269 (91.5%)	
Yes	179 (8.1%)	154 (8.1%)	25 (8.5%)	
Surgery				<0.001
No	2006 (91.1%)	1756 (92.0%)	250 (85.0%)	
Yes	196 (8.9%)	152 (8.0%)	44 (15.0%)	
CRRT				<0.001
No	2,154 (97.8%)	1886 (98.8%)	268 (91.2%)	
Yes	48 (2.2%)	22 (1.2%)	26 (8.8%)	
Mechanical ventilation				<0.001
No	657 (29.8%)	611 (32.0%)	46 (15.6%)	
Yes	1,545 (70.2%)	1,297 (68.0%)	248 (84.4%)	

*N* = 2,202, including 1,908 hospital survivors and 294 non-survivors. Continuous variables are median (IQR); categorical variables are *n* (%). Platelet count CV was calculated using only nonmissing pre-ICU platelet values. GCS, Glasgow Coma Scale; SOFA, Sequential Organ Failure Assessment:; APSIII, Acute Physiology Score III; SAPSII, Simplified Acute Physiology Score II; HR, Heart Rate; RR, Respiratory Rate; NBPS, Non-invasive Blood Pressure Systolic; NBPD, Non-invasive Blood Pressure Diastolic; RDW, Red Cell Distribution Width; RBC, Red Blood Cell; WBC, White Blood Cell; AG, Anion Gap; PT, Prothrombin Time; PTT, Partial Thromboplastin Time; ALT, Alanine Aminotransferase; AST, Aspartate Aminotransferase; TBIL, Total Bilirubin; SLE, Systemic Lupus Erythematosus; ALL, Acute Lymphoblastic Leukemia; MDS, Myelodysplastic Syndrome; ITP, Immune Thrombocytopenic Purpura; TP, Thrombocytopenia; AKI, Acute Kidney Injury; HTN, Hypertension; LC, Liver Cirrhosis; HEP, Hepatitis; PNA, Pneumonia; CKD, Chronic Kidney Disease; CA, Carcinoma; T2DM, Type 2 Diabetes Mellitus; HLD, Hyperlipidemia; HF, Heart Failure; MI, Myocardial Infarction; COPD, Chronic Obstructive Pulmonary Disease; Anti-coa, Anticoagulation agent; Anti-pla, Antiplatelet agent; CRRT, Continuous Renal Replacement Therapy; Platelet count CV, Coefficient of Variation of Platelet Count.

### 3.2 Association between platelet count CV and in-hospital mortality

**Figure 3:**
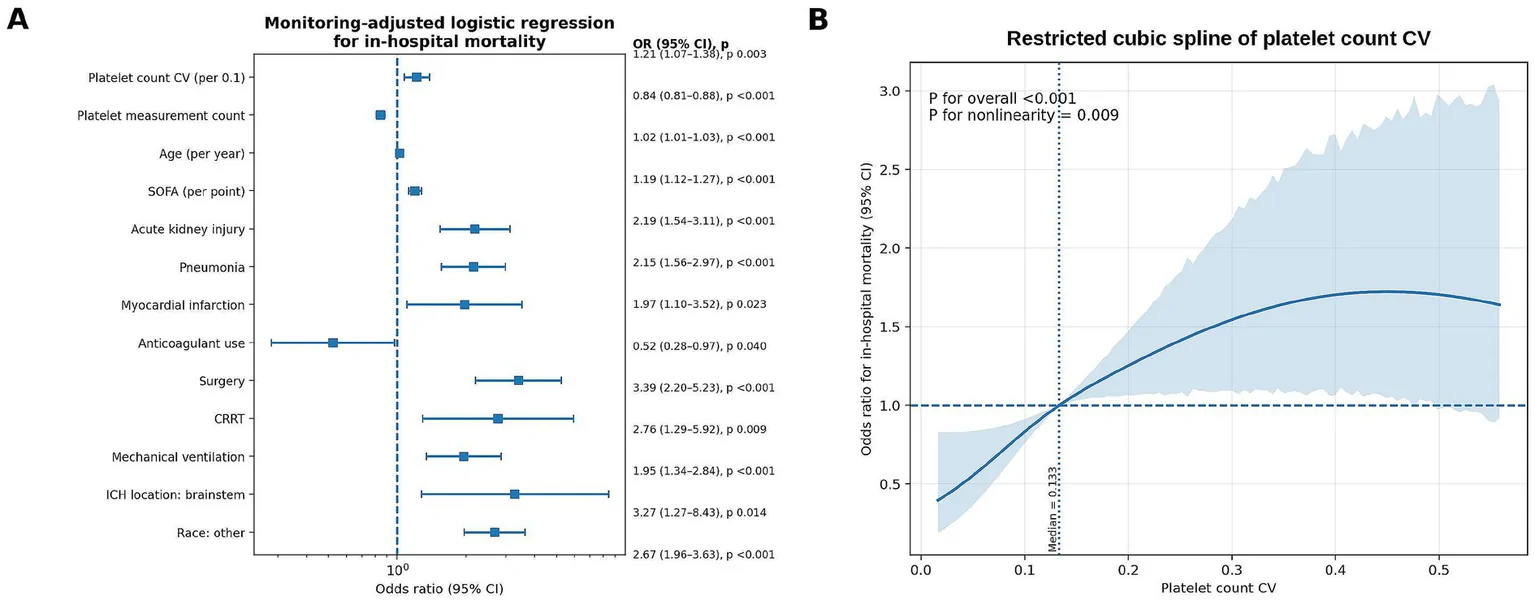
**(A)** Forest plot of the monitoring-adjusted multivariable logistic regression model for in-hospital mortality. The *OR* for platelet count *CV* is reported per 0.1-unit increase. **(B)** Restricted cubic spline analysis of platelet count *CV* and in-hospital mortality, adjusted using the same covariates as the primary model, including platelet measurement count. CV, coefficient of variation; CI, confidence interval; OR, odds ratio.

In univariable logistic regression, each 0.1-unit increase in platelet count CV was associated with higher in-hospital mortality (OR, 1.28; 95% CI, 1.17–1.40; *p* < 0.001). In the multivariable model without platelet measurement count, the association was attenuated and was not statistically significant (OR, 0.99; 95% CI, 0.88–1.11; *p* = 0.901). After additional adjustment for platelet measurement count, each 0.1-unit increase in CV was associated with higher in-hospital mortality (OR, 1.21; 95% CI, 1.07–1.38; *p* = 0.003; Table 2; Figure 3A). Platelet measurement count was inversely associated with in-hospital mortality (OR per additional measurement, 0.84; 95% CI, 0.81–0.88; *p* < 0.001).

**Table 2:** Logistic regression analyses for in-hospital mortality.

Variable	Univariate OR (95% CI)	Univariate *p*	Adjusted OR without count (95% CI)	Adjusted *p* without count	Monitoring-adjusted OR (95% CI)	Monitoring-adjusted *p*
Platelet count CV, per 0.1	1.28 (1.17–1.40)	<0.001	0.99 (0.88–1.11)	0.901	1.21 (1.07–1.38)	0.003
Platelet measurement count, per measurement	0.97 (0.95–1.00)	0.061	—	—	0.84 (0.81–0.88)	<0.001
Age, per year	1.01 (1.00–1.02)	0.014	1.02 (1.01–1.04)	<0.001	1.02 (1.01–1.03)	<0.001
Male (vs female)	1.06 (0.83–1.35)	0.664	0.96 (0.73–1.26)	0.761	0.97 (0.73–1.28)	0.823
GCS, per point	0.95 (0.91–0.99)	0.009	1.05 (1.00–1.10)	0.054	1.04 (0.99–1.10)	0.096
SOFA, per point	1.26 (1.21–1.32)	<0.001	1.21 (1.13–1.29)	<0.001	1.19 (1.12–1.27)	<0.001
Baseline platelet count, per 10 K/μL	0.97 (0.95–0.98)	<0.001	0.99 (0.97–1.01)	0.395	1.00 (0.98–1.02)	0.851
AKI (yes vs. no)	2.92 (2.24–3.80)	<0.001	1.74 (1.24–2.42)	0.001	2.19 (1.54–3.11)	<0.001
Pneumonia (yes vs. no)	2.31 (1.78–3.00)	<0.001	1.50 (1.11–2.01)	0.008	2.15 (1.56–2.97)	<0.001
CKD (yes vs. no)	1.52 (1.09–2.12)	0.014	0.98 (0.66–1.46)	0.918	0.99 (0.65–1.49)	0.945
Myocardial infarction (yes vs. no)	2.72 (1.68–4.40)	<0.001	1.73 (1.00–3.02)	0.051	1.97 (1.10–3.52)	0.023
Anticoagulant use (yes vs. no)	0.57 (0.34–0.97)	0.037	0.39 (0.22–0.71)	0.002	0.52 (0.28–0.97)	0.040
Surgery (yes vs. no)	2.03 (1.42–2.92)	<0.001	2.67 (1.76–4.05)	<0.001	3.39 (2.20–5.23)	<0.001
CRRT (yes vs. no)	8.32 (4.65–14.88)	<0.001	2.56 (1.23–5.31)	0.012	2.76 (1.29–5.92)	0.009
Mechanical ventilation (yes vs. no)	2.54 (1.83–3.53)	<0.001	1.49 (1.03–2.14)	0.032	1.95 (1.34–2.84)	<0.001
Platelet transfusion (yes vs. no)	2.68 (1.76–4.08)	<0.001	1.19 (0.70–2.02)	0.524	1.22 (0.70–2.12)	0.490
ICH location: Cerebral lobe (vs deep hemorrhage)	0.78 (0.55–1.10)	0.160	0.79 (0.42–1.48)	0.465	0.73 (0.38–1.38)	0.333
ICH location: Brainstem (vs deep hemorrhage)	2.28 (1.14–4.57)	0.020	3.52 (1.40–8.84)	0.008	3.27 (1.27–8.43)	0.014
ICH location: Cerebellum (vs deep hemorrhage)	0.94 (0.53–1.68)	0.843	0.90 (0.41–1.99)	0.790	0.88 (0.38–2.02)	0.763
ICH location: Ventricle (vs deep hemorrhage)	1.63 (1.16–2.29)	0.004	1.69 (0.91–3.13)	0.096	1.83 (0.96–3.47)	0.065
ICH location: Multifocal hemorrhage (vs deep hemorrhage)	1.53 (0.51–4.59)	0.444	1.73 (0.49–6.14)	0.395	1.86 (0.51–6.76)	0.349
ICH location: Others (vs deep hemorrhage)	0.89 (0.70–1.14)	0.354	1.10 (0.63–1.93)	0.727	1.00 (0.56–1.77)	0.997
Race: Black (vs White)	0.86 (0.56–1.32)	0.481	1.17 (0.72–1.91)	0.519	1.30 (0.80–2.13)	0.294
Race: Asian (vs White)	0.90 (0.47–1.71)	0.748	1.61 (0.81–3.23)	0.176	1.81 (0.89–3.68)	0.099
Race: Others (vs White)	2.43 (1.88–3.13)	<0.001	2.45 (1.82–3.30)	<0.001	2.67 (1.96–3.63)	<0.001

Platelet count CV is reported per 0.1-unit increase. The monitoring-adjusted model additionally included platelet measurement count. *N* = 2,202; events = 294. GCS, Glasgow Coma Scale; SOFA, Sequential Organ Failure Assessment:; APSIII, Acute Physiology Score III; SAPSII, Simplified Acute Physiology Score II; HR, Heart Rate; RR, Respiratory Rate; NBPS, Non-invasive Blood Pressure Systolic; NBPD, Non-invasive Blood Pressure Diastolic; RDW, Red Cell Distribution Width; RBC, Red Blood Cell; WBC, White Blood Cell; AG, Anion Gap; PT, Prothrombin Time; PTT, Partial Thromboplastin Time; ALT, Alanine Aminotransferase; AST, Aspartate Aminotransferase; TBIL, Total Bilirubin; SLE, Systemic Lupus Erythematosus; ALL, Acute Lymphoblastic Leukemia; MDS, Myelodysplastic Syndrome; ITP, Immune Thrombocytopenic Purpura; TP, Thrombocytopenia; AKI, Acute Kidney Injury; HTN, Hypertension; LC, Liver Cirrhosis; HEP, Hepatitis; PNA, Pneumonia; CKD, Chronic Kidney Disease; CA, Carcinoma; T2DM, Type 2 Diabetes Mellitus; HLD, Hyperlipidemia; HF, Heart Failure; MI, Myocardial Infarction; COPD, Chronic Obstructive Pulmonary Disease; Anti-coa, Anticoagulation agent; Anti-pla, Antiplatelet agent; CRRT, Continuous Renal Replacement Therapy; Platelet count CV, Coefficient of Variation of Platelet Count.

The monitoring-adjusted restricted cubic spline analysis showed an overall association between platelet count CV and in-hospital mortality (*p* for overall association <0.001), with evidence of nonlinearity (*p* for nonlinearity = 0.009; Figure 3B).

### 3.3 Survival analysis

**Figure 4:**
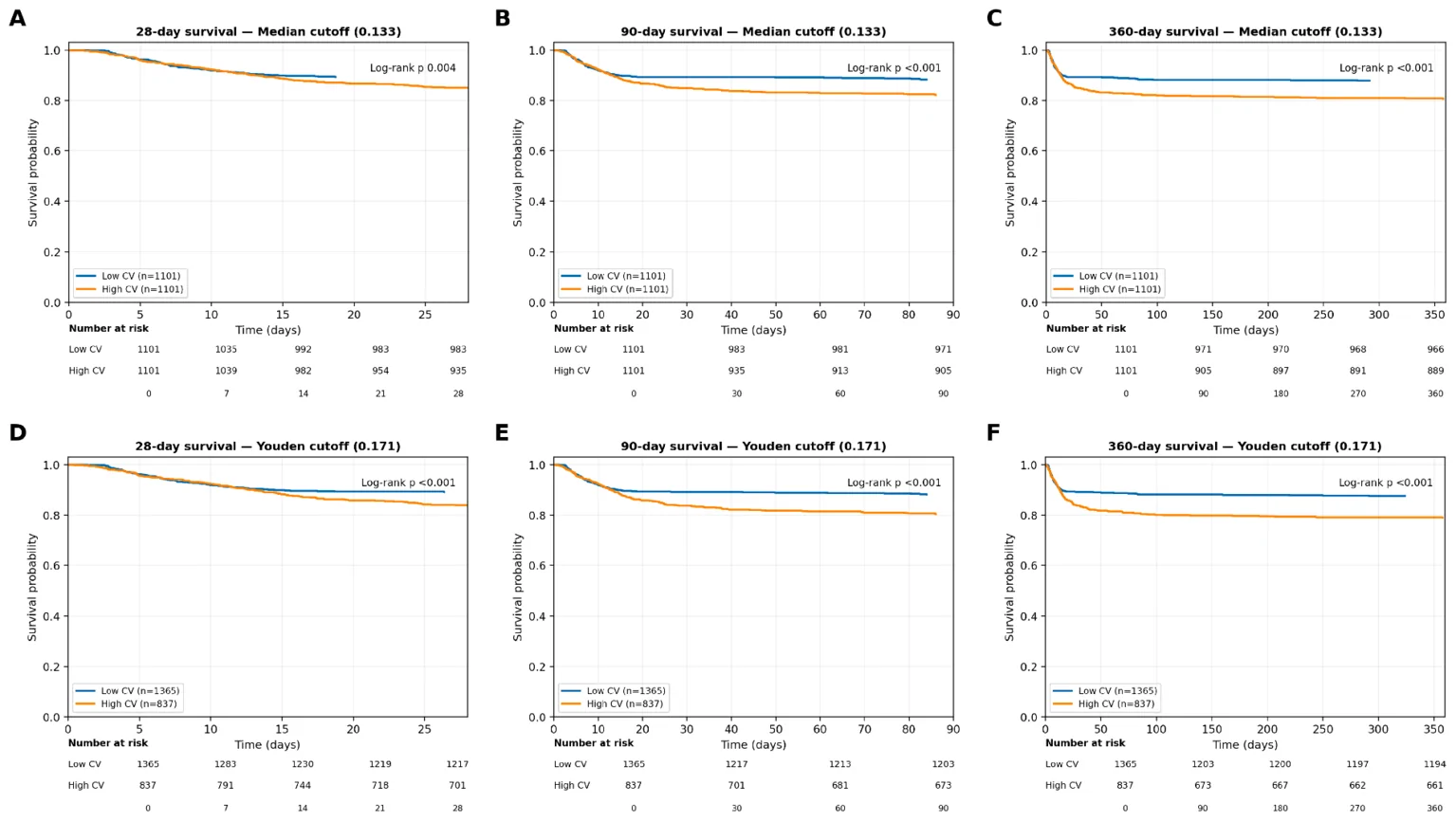
**(A–C)** Kaplan–Meier curves for 28-day, 90-day, and 360-day survival using the median platelet count CV cutoff of 0.133. **(D–F)** Corresponding survival curves using the Youden-index-derived cutoff of 0.171. CV, coefficient of variation.

Patients were stratified using the median CV of 0.133 and the Youden-index-derived cutoff of 0.171. With the median cutoff, 1,101 patients were assigned to each group. With the Youden cutoff, 1,365 patients were classified as low CV and 837 as high CV. High-CV patients had lower survival at 28 days (log-rank *p* = 0.004), 90 days (*p* < 0.001), and 360 days (*p* < 0.001) using the median cutoff; all three comparisons were also significant using the Youden cutoff (all *p* < 0.001; Figure 4).

In monitoring-adjusted multivariable Cox models, each 0.1-unit increase in platelet count CV was associated with 28-day mortality (HR, 1.14; 95% CI, 1.04–1.26; *p* = 0.006), 90-day mortality (HR, 1.11; 95% CI, 1.02–1.21; *p* = 0.014), and 360-day mortality (HR, 1.11; 95% CI, 1.02–1.20; *p* = 0.016; Table 3; Supplementary Figure S1). Univariable Cox results are presented in Supplementary Table S1.

**Table 3:** Monitoring-adjusted Cox proportional hazards models.

Variable	28-day HR (95% CI)	*p*-value (28-day)	90-day HR (95% CI)	*p*-value (90-day)	360-day HR (95% CI)	*p*-value (360-day)
Platelet count CV, per 0.1	1.14 (1.04–1.26)	0.006	1.11 (1.02–1.21)	0.014	1.11 (1.02–1.20)	0.016
Platelet measurement count, per measurement	0.82 (0.79–0.85)	<0.001	0.86 (0.83–0.89)	<0.001	0.87 (0.84–0.89)	<0.001
Age, per year	1.02 (1.01–1.03)	<0.001	1.01 (1.01–1.02)	<0.001	1.01 (1.01–1.02)	0.001
Male (vs female)	0.93 (0.73–1.19)	0.553	0.92 (0.73–1.16)	0.480	0.94 (0.76–1.18)	0.611
GCS, per point	1.03 (0.98–1.07)	0.220	1.02 (0.98–1.06)	0.314	1.03 (0.99–1.07)	0.179
SOFA, per point	1.16 (1.10–1.22)	<0.001	1.15 (1.10–1.21)	<0.001	1.15 (1.10–1.21)	<0.001
Baseline platelet count, per 10 K/μL	1.00 (0.99–1.02)	0.540	1.00 (0.99–1.02)	0.607	1.00 (0.99–1.02)	0.812
AKI (yes vs. no)	1.79 (1.32–2.44)	<0.001	1.77 (1.33–2.35)	<0.001	1.78 (1.35–2.35)	<0.001
Pneumonia (yes vs. no)	2.03 (1.54–2.66)	<0.001	2.01 (1.56–2.58)	<0.001	1.96 (1.53–2.50)	<0.001
CKD (yes vs. no)	1.08 (0.76–1.52)	0.669	1.06 (0.76–1.46)	0.740	1.00 (0.73–1.37)	0.977
Myocardial infarction (yes vs. no)	1.62 (1.02–2.56)	0.042	1.70 (1.11–2.59)	0.015	1.81 (1.21–2.71)	0.004
Anticoagulant use (yes vs. no)	0.51 (0.29–0.91)	0.023	0.61 (0.38–0.99)	0.043	0.67 (0.43–1.04)	0.076
Surgery (yes vs. no)	2.79 (1.93–4.02)	<0.001	2.71 (1.93–3.80)	<0.001	2.51 (1.80–3.50)	<0.001
CRRT (yes vs. no)	1.36 (0.75–2.48)	0.312	1.64 (0.96–2.81)	0.068	1.58 (0.94–2.64)	0.082
Mechanical ventilation (yes vs. no)	1.79 (1.28–2.49)	<0.001	1.73 (1.26–2.37)	<0.001	1.67 (1.23–2.26)	<0.001
Platelet transfusion (yes vs. no)	1.31 (0.85–2.02)	0.227	1.29 (0.86–1.92)	0.222	1.28 (0.87–1.89)	0.218
ICH location: Cerebral lobe (vs deep hemorrhage)	0.62 (0.36–1.09)	0.095	0.63 (0.37–1.06)	0.083	0.67 (0.40–1.12)	0.125
ICH location: Brainstem (vs deep hemorrhage)	2.02 (0.93–4.39)	0.077	2.13 (1.02–4.45)	0.044	2.09 (1.01–4.33)	0.048
ICH location: Cerebellum (vs deep hemorrhage)	0.70 (0.34–1.44)	0.332	0.80 (0.41–1.55)	0.508	0.78 (0.41–1.50)	0.458
ICH location: Ventricle (vs deep hemorrhage)	1.43 (0.82–2.47)	0.204	1.46 (0.87–2.46)	0.148	1.38 (0.83–2.29)	0.213
ICH location: Multifocal hemorrhage (vs deep hemorrhage)	1.77 (0.65–4.80)	0.264	1.93 (0.77–4.84)	0.162	1.83 (0.73–4.56)	0.197
ICH location: Others (vs deep hemorrhage)	0.83 (0.51–1.36)	0.464	0.87 (0.55–1.39)	0.560	0.91 (0.58–1.44)	0.698
Race: Black (vs White)	1.19 (0.76–1.87)	0.442	1.13 (0.74–1.71)	0.578	1.18 (0.80–1.75)	0.396
Race: Asian (vs White)	1.68 (0.88–3.23)	0.117	1.91 (1.10–3.34)	0.022	1.89 (1.10–3.22)	0.020
Race: Others (vs White)	2.45 (1.88–3.18)	<0.001	2.12 (1.65–2.71)	<0.001	1.98 (1.56–2.52)	<0.001

Platelet count CV is reported per 0.1-unit increase. All models included platelet measurement count. Events: 28-day = 284; 90-day = 326; 360-day = 347.

### 3.4 Subgroup analyses

**Figure 5:**
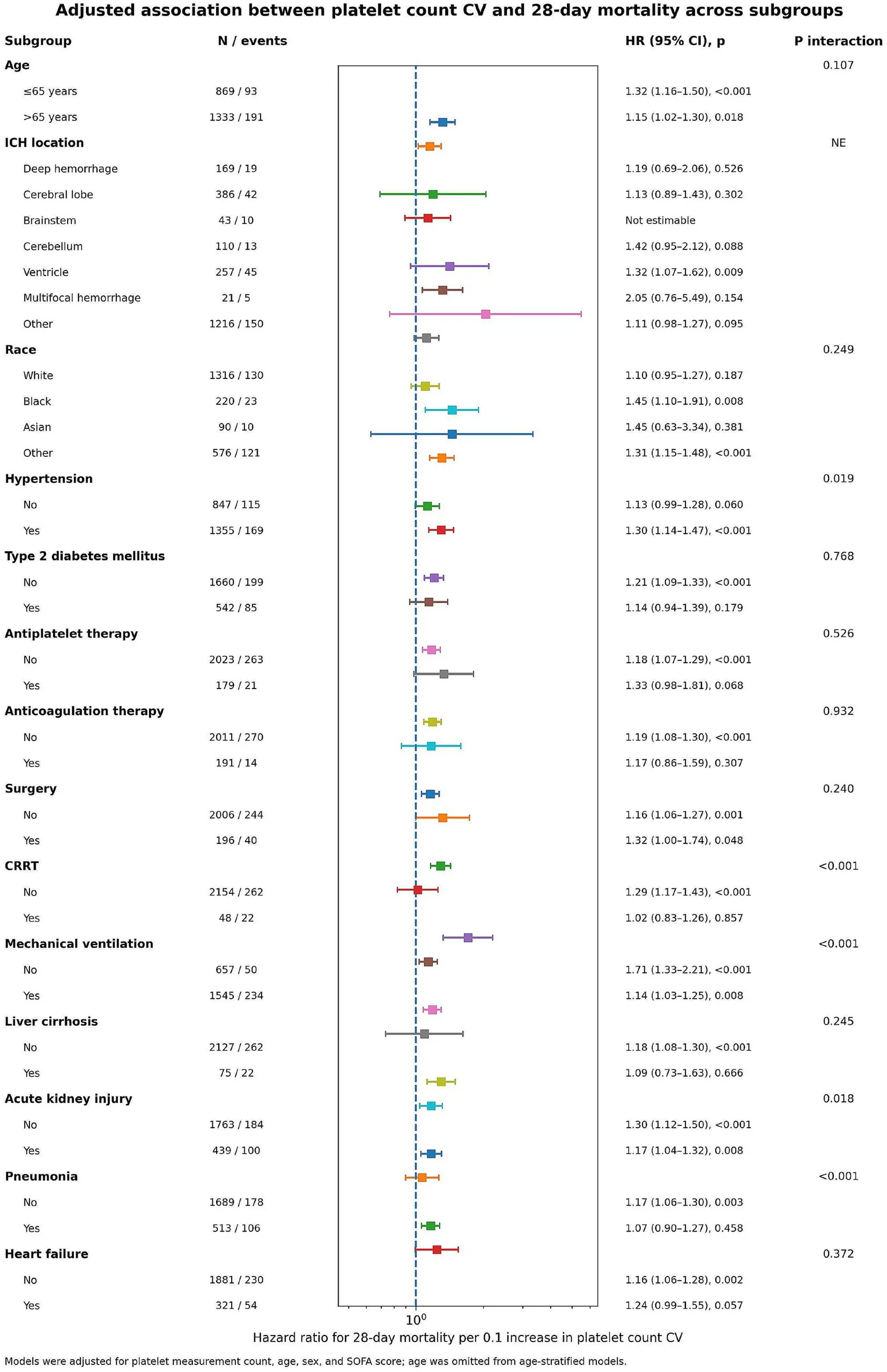
Subgroup analyses of the association between platelet count CV and 28-day mortality. HRs are reported per 0.1-unit increase. Models were adjusted for platelet measurement count, age, sex, and SOFA score, except that age was omitted from age-stratified models. Interaction *p* values were obtained from product terms between CV and the subgroup variable. CI, confidence interval; CRRT, continuous renal replacement therapy; CV, coefficient of variation; HR, hazard ratio.

The association between platelet count CV and 28-day mortality was evaluated across prespecified subgroups, with HRs reported per 0.1-unit increase (Figure 5). Exploratory interaction tests suggested heterogeneity by hypertension (*p* for interaction = 0.019), CRRT use (*p* < 0.001), mechanical ventilation (*p* < 0.001), acute kidney injury (*p* = 0.018), and pneumonia (*p* < 0.001). The association was stronger among patients with hypertension and among those without CRRT, mechanical ventilation, acute kidney injury, or pneumonia. The interaction for ICH location was not estimable because of sparse events in some categories. Other interaction tests were not statistically significant. Because multiple exploratory interactions were examined without multiplicity adjustment, these findings should be interpreted cautiously.

### 3.5 Incremental prognostic value of platelet count CV

**Figure 6:**
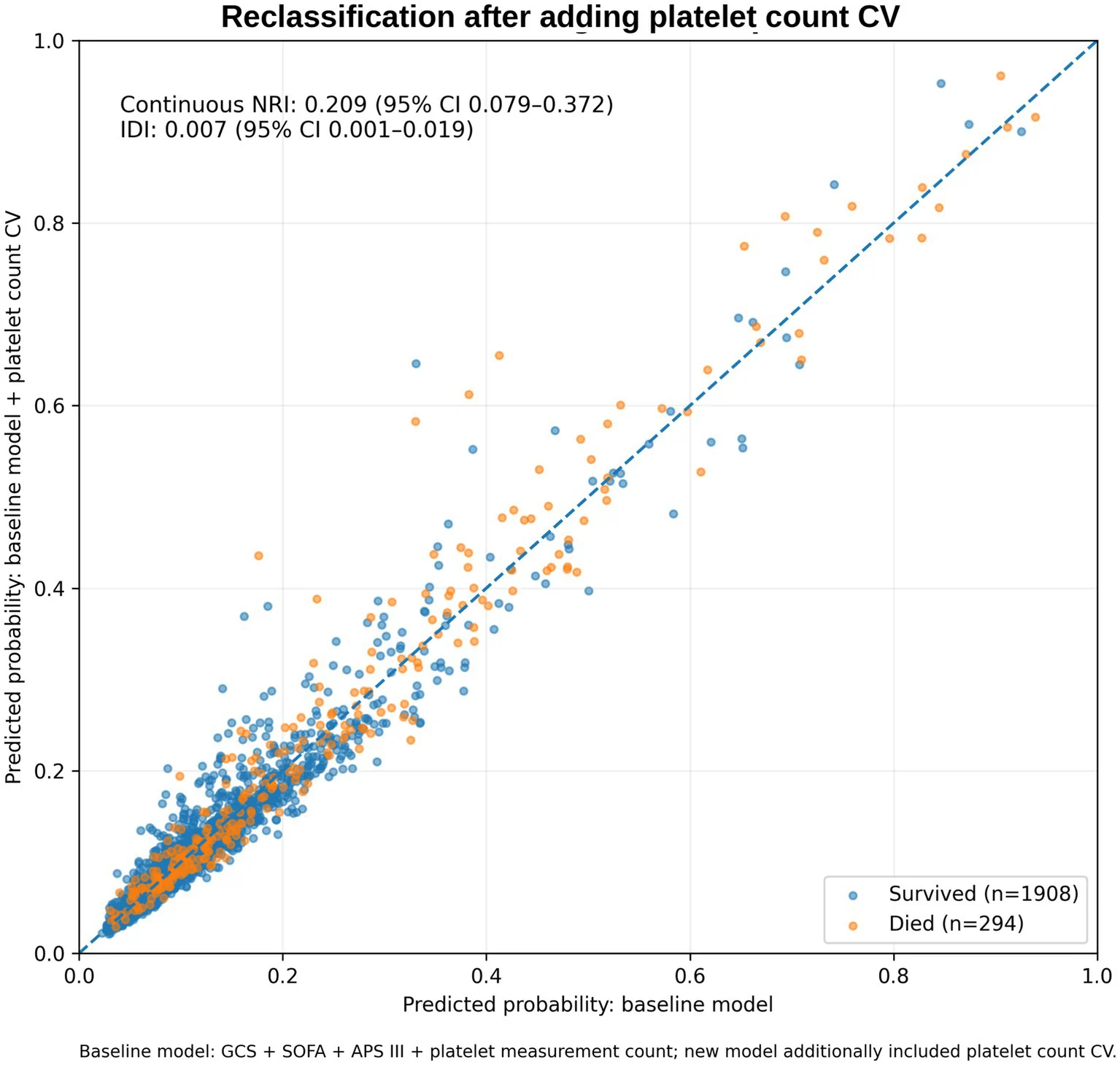
Reclassification after adding platelet count CV to the baseline prognostic model. The baseline model included GCS, SOFA, APS III, and platelet measurement count; the extended model additionally included platelet count CV. The continuous NRI was 0.209 (95% CI, 0.079–0.372), and the IDI was 0.007 (95% CI, 0.001–0.019). CV, coefficient of variation; IDI, integrated discrimination improvement; NRI, net reclassification improvement.

Adding platelet count CV to a baseline model containing GCS, SOFA, APS III, and platelet measurement count produced a modest improvement in risk classification. The continuous NRI was 0.209 (95% CI, 0.079–0.372), and the IDI was 0.007 (95% CI, 0.001–0.019; Figure 6). These results indicate limited incremental prognostic information rather than a major improvement in predictive performance.

### 3.6 Sensitivity analyses

Among the 761 patients excluded for having fewer than three eligible pre-ICU platelet measurements, patient-level covariates were available for 517 patients with one or two measurements. Compared with the 2,202 included patients, these 517 excluded patients were older and had markedly higher in-hospital mortality (42.9% vs. 13.4%) and 28-day mortality (43.9% vs. 12.9%), while also differing in several measures of illness severity and treatment intensity (Supplementary Table S6). These differences indicate substantial selection related to the platelet-monitoring process.

In clinically adjusted models that used the fixed last three pre-ICU platelet measurements, each 0.1-unit increase in CV was associated with in-hospital mortality (OR, 1.33; 95% CI, 1.15–1.53; *p* < 0.001), 28-day mortality (HR, 1.25; 95% CI, 1.14–1.38; *p* < 0.001), 90-day mortality (HR, 1.20; 95% CI, 1.09–1.32; *p* < 0.001), and 360-day mortality (HR, 1.18; 95% CI, 1.07–1.29; *p* < 0.001). After additional adjustment for total platelet measurement count, the corresponding estimates remained significant: OR 1.26 (95% CI, 1.09–1.46; *p* = 0.002) for in-hospital mortality and HRs of 1.22 (95% CI, 1.11–1.36; *p* < 0.001), 1.17 (95% CI, 1.06–1.29; *p* = 0.001), and 1.15 (95% CI, 1.05–1.27; *p* = 0.004) for 28-day, 90-day, and 360-day mortality, respectively (Supplementary Table S7).

The ICU-period landmark analyses were also directionally consistent. In the 3-day landmark cohort (*N* = 2,088), CV was associated with 28-day mortality (HR, 1.42; 95% CI, 1.25–1.61, *p* < 0.001), 90-day mortality (HR, 1.34; 95% CI, 1.17–1.56, *p* < 0.001), and 360-day mortality (HR, 1.27; 95% CI, 1.12–1.44; *p* < 0.001). In the 7-day landmark cohort (*N* = 1,993), the corresponding HRs were 1.39 (95% CI, 1.14–1.69, *p* < 0.001), 1.27 (95% CI, 1.11–1.45, *p* < 0.001), and 1.14 (95% CI, 1.02–1.29, *p* = 0.024), respectively (Supplementary Table S4).

Complete admission CT imaging data were available for all 281 patients in the external validation cohort. At 28 days, 229 patients were alive and 52 had died. Median hematoma volume was significantly higher among patients who died within 28 days than among those who survived (47.32 mL [IQR, 28.90–63.54] vs. 30.21 mL [IQR, 6.24–47.21]; *p* < 0.001). In the external-cohort sensitivity analysis with additional adjustment for hematoma volume and IVH, platelet count CV remained associated with 28-day mortality (HR, 1.37; 95% CI, 1.12–1.59; *p* < 0.001), 90-day mortality (HR, 1.26; 95% CI, 1.07–1.49; *p* = 0.006), and 360-day mortality (HR, 1.17; 95% CI, 1.02–1.32; *p* = 0.017; Supplementary Table S5). Residual confounding by other unavailable ICH-specific factors could not be excluded.

### 3.7 External validation

The external validation cohort included 281 patients (Supplementary Table S2), and univariable Cox results are presented in Supplementary Table S3. Using the Youden-index-derived cutoff, the high-CV group had lower survival at 28 days (log-rank *p* = 0.011), 90 days (*p* = 0.010), and 360 days (*p* = 0.012; Figures 2A–C). Restricted cubic spline analysis showed an overall association with 28-day mortality (*p* < 0.001) and evidence of nonlinearity (*p* = 0.007; Figure 2D).

Platelet count CV yielded an AUC of 0.786 (95% CI, 0.717–0.856) for 28-day mortality, with sensitivity of 0.792 and specificity of 0.672 (Figure 2E). In the external multivariable Cox model, each 0.1-unit increase in CV was associated with 28-day mortality (HR, 1.141; 95% CI, 1.007–1.292; *p* = 0.042; Figure 2F).

## 4 Discussion

In this multicenter retrospective study, pre-ICU platelet count CV was associated with in-hospital and longer-term mortality after adjustment for clinical covariates and platelet-monitoring frequency. The magnitude of association was modest when expressed per 0.1-unit increase. Findings were supported by fixed-three-measurement and ICU-period landmark analyses and were directionally reproduced in the external cohort. However, the association was sensitive to adjustment for monitoring frequency, and patients excluded because of insufficient platelet measurements differed substantially from those included. These findings support CV as a potential dynamic prognostic marker, but not as a proven causal factor or therapeutic target.

Previous neurocritical-care studies have mainly focused on thrombocytopenia and static platelet counts (6, 9–11). Greater platelet variability may reflect systemic inflammation, endothelial dysfunction, bleeding progression, infection, transfusion exposure, organ dysfunction, treatment intensity, or other sources of clinical instability (5, 14–17). Because these factors may influence both platelet trajectories and mortality, the observed association should not be interpreted as evidence that platelet fluctuation itself directly causes poor outcomes.

Platelet-monitoring frequency was a particularly important methodological consideration. Measurement count was lower among non-survivors and was strongly associated with outcomes. Moreover, the CV association was attenuated in models that did not include measurement count and became apparent after monitoring-frequency adjustment. This pattern is compatible with confounding or suppression by the clinical testing process, including short pre-ICU observation, early deterioration, or early death. The consistent fixed-three-measurement analysis reduced, but did not eliminate, this concern because exact measurement timing and observation-window duration were unavailable.

From a clinical perspective, platelet count CV is readily calculated from routine complete blood counts and may provide supplementary information regarding patient instability. Nevertheless, the incremental prediction analyses showed only modest improvement, particularly for the IDI. The median and Youden-derived cutoffs were data-driven and should not be considered validated clinical thresholds. The present results also do not support interventions intended specifically to stabilize platelet trajectories or routine platelet transfusion on the basis of CV alone (12, 18, 19).

The external cohort provided directional support for the primary findings, with significant survival differences, a nonlinear association, moderate discrimination, and a modest adjusted association with 28-day mortality. All 281 external-cohort patients had complete admission CT imaging data, and hematoma volume was higher among 28-day non-survivors than survivors. Additional adjustment for hematoma volume and IVH did not eliminate the association. However, the external sample and event numbers were limited, and the model included numerous covariates. External validation and imaging-adjusted results should therefore be viewed as supportive rather than definitive.

Future prospective studies should use predefined platelet-sampling schedules, preserve exact measurement timestamps, and collect detailed ICH-specific variables, including hematoma volume, IVH burden, hematoma expansion, formal ICH scores, and treatment-limitation decisions. Such studies are needed to determine whether CV adds clinically meaningful information beyond established severity measures such as GCS and SOFA (20).

## 5 Limitations

This study has several limitations. First, its retrospective observational design precludes causal inference and leaves the possibility of residual confounding. Second, platelet count CV depends on the frequency and timing of laboratory testing. The estimated association changed materially after adjustment for measurement count, indicating that monitoring intensity was an important component of the observed relationship. Third, requiring at least three measurements introduced selection: patients excluded for insufficient measurements had substantially higher mortality and differed in clinical characteristics from those included. The comparison in Supplementary Table S6 included 517 excluded patients with available patient-level covariates; corresponding covariates were unavailable for the remaining 244 patients excluded for having fewer than three eligible measurements. Patients admitted directly to the ICU may have had insufficient opportunity to accumulate three pre-ICU platelet measurements; therefore, the findings may not be generalizable to this group. Fourth, exact sampling timestamps and the interval between the first and last eligible measurements were unavailable, preventing adjustment for observation-window duration. Exact ICH symptom-onset time was also not reliably available. Fifth, although complete admission CT imaging data and report-based hematoma-volume estimates were available in the external cohort, several other ICH-specific prognostic factors, including hematoma expansion, formal ICH score, IVH burden beyond binary status, and withdrawal-of-care decisions, were incomplete or unavailable. Sixth, the external validation cohort was relatively small, and the number of events limited the reliability of extensive multivariable adjustment. Finally, the derived cutoffs were exploratory and require prospective validation.

## 6 Conclusion

Higher pre-ICU platelet count CV was associated with in-hospital and longer-term mortality after adjustment for clinical covariates and platelet-monitoring frequency. The association was directionally reproduced in an independent external cohort, although the external effect estimate was modest. Platelet count CV may provide supplementary prognostic information, but monitoring-related selection, residual confounding, and the retrospective design limit immediate clinical application. Prospective studies using standardized platelet-sampling schedules are required before platelet count CV can be adopted as a clinical risk marker.

## Data Availability

The original contributions presented in the study are included in the article/Supplementary material, further inquiries can be directed to the corresponding author.
