# Supplementary material for: Coefficient of variation of platelet count as a prognostic Indicator for intracerebral hemorrhage: findings from a multicenter cohort
Source: Front Neurol. 2026 Aug 19;17:1868807. doi: 10.3389/fneur.2026.1868807 (PMC13533765; doi:10.3389/fneur.2026.1868807)
Supplement: Supplementary file 1 [file Table_1.docx]

**Table S1. Univariable Cox proportional hazards models in the primary cohort**

| **Variable** | **28-day HR (95% CI)** | ***p*-value(28-day)** | **90-day HR (95% CI)** | ***p*-value(90-day)** | **360-day HR (95% CI)** | ***p*-value(360-day)** |
| --- | --- | --- | --- | --- | --- | --- |
| Platelet count CV, per 0.1 | 1.17 (1.08–1.27) | <0.001 | 1.21 (1.13–1.30) | <0.001 | 1.22 (1.14–1.31) | <0.001 |
| Platelet measurement count, per measurement | 0.94 (0.92–0.97) | <0.001 | 0.97 (0.95–1.00) | 0.044 | 0.98 (0.96–1.01) | 0.127 |
| Age, per year | 1.01 (1.01–1.02) | 0.001 | 1.01 (1.00–1.02) | 0.005 | 1.01 (1.00–1.02) | 0.011 |
| Male (vs female) | 1.00 (0.79–1.26) | 0.992 | 0.99 (0.80–1.23) | 0.936 | 1.02 (0.83–1.26) | 0.839 |
| GCS, per point | 0.95 (0.91–0.99) | 0.006 | 0.94 (0.91–0.98) | 0.001 | 0.95 (0.92–0.98) | 0.003 |
| SOFA, per point | 1.20 (1.16–1.24) | <0.001 | 1.20 (1.17–1.24) | <0.001 | 1.21 (1.17–1.24) | <0.001 |
| Baseline platelet count, per 10 K/μL | 0.98 (0.96–0.99) | 0.002 | 0.98 (0.96–0.99) | 0.002 | 0.97 (0.96–0.99) | <0.001 |
| AKI (yes vs no) | 2.30 (1.81–2.94) | <0.001 | 2.44 (1.95–3.06) | <0.001 | 2.50 (2.01–3.11) | <0.001 |
| Pneumonia (yes vs no) | 2.03 (1.60–2.58) | <0.001 | 2.17 (1.74–2.72) | <0.001 | 2.16 (1.74–2.68) | <0.001 |
| CKD (yes vs no) | 1.41 (1.03–1.92) | 0.032 | 1.42 (1.06–1.90) | 0.020 | 1.38 (1.03–1.83) | 0.029 |
| Myocardial infarction (yes vs no) | 2.29 (1.50–3.51) | <0.001 | 2.38 (1.61–3.53) | <0.001 | 2.52 (1.73–3.66) | <0.001 |
| Anticoagulant use (yes vs no) | 0.52 (0.31–0.89) | 0.018 | 0.66 (0.42–1.03) | 0.069 | 0.75 (0.49–1.13) | 0.168 |
| Surgery (yes vs no) | 1.74 (1.24–2.43) | 0.001 | 1.80 (1.32–2.45) | <0.001 | 1.72 (1.27–2.34) | <0.001 |
| CRRT (yes vs no) | 4.38 (2.83–6.77) | <0.001 | 4.94 (3.33–7.33) | <0.001 | 5.12 (3.50–7.49) | <0.001 |
| Mechanical ventilation (yes vs no) | 2.04 (1.51–2.77) | <0.001 | 2.17 (1.62–2.90) | <0.001 | 2.12 (1.60–2.80) | <0.001 |
| Platelet transfusion (yes vs no) | 2.42 (1.68–3.50) | <0.001 | 2.48 (1.76–3.49) | <0.001 | 2.54 (1.83–3.53) | <0.001 |
| ICH location: Cerebral lobe (vs deep hemorrhage) | 0.80 (0.58–1.12) | 0.194 | 0.76 (0.56–1.04) | 0.084 | 0.80 (0.59–1.07) | 0.132 |
| ICH location: Brainstem (vs deep hemorrhage) | 2.00 (1.06–3.75) | 0.032 | 1.92 (1.05–3.51) | 0.033 | 1.81 (0.99–3.29) | 0.053 |
| ICH location: Cerebellum (vs deep hemorrhage) | 0.90 (0.51–1.56) | 0.699 | 0.97 (0.58–1.60) | 0.892 | 0.90 (0.55–1.49) | 0.696 |
| ICH location: Ventricle (vs deep hemorrhage) | 1.47 (1.07–2.03) | 0.017 | 1.49 (1.11–2.01) | 0.008 | 1.39 (1.03–1.87) | 0.029 |
| ICH location: Multifocal hemorrhage (vs deep hemorrhage) | 1.86 (0.77–4.51) | 0.168 | 1.98 (0.88–4.45) | 0.096 | 1.88 (0.84–4.21) | 0.126 |
| ICH location: Others (vs deep hemorrhage) | 0.90 (0.71–1.14) | 0.384 | 0.92 (0.74–1.14) | 0.458 | 0.96 (0.78–1.19) | 0.722 |
| Race: Black (vs White) | 0.78 (0.51–1.20) | 0.255 | 0.80 (0.54–1.18) | 0.264 | 0.87 (0.60–1.25) | 0.450 |
| Race: Asian (vs White) | 0.86 (0.46–1.61) | 0.636 | 1.05 (0.62–1.80) | 0.850 | 1.06 (0.63–1.78) | 0.822 |
| Race: Others (vs White) | 2.23 (1.76–2.82) | <0.001 | 1.98 (1.59–2.48) | <0.001 | 1.87 (1.50–2.32) | <0.001 |

**Note:** Platelet count CV is reported per 0.1-unit increase. N=2,202. GCS, Glasgow Coma Scale; SOFA, Sequential Organ Failure Assessment; APS III, Acute Physiology Score III; SAPS II, Simplified Acute Physiology Score II; AKI, acute kidney injury; CKD, chronic kidney disease; CRRT, continuous renal replacement therapy; CI, confidence interval; HR, hazard ratio.

**Table S2. Baseline characteristics of the external validation cohort**

| **Variable Names** | **level** | **Overall N=281** | **Non-Death N=229** | **Death N=52** | **p** |
| --- | --- | --- | --- | --- | --- |
| Hematocrit |  | 37.9 (32.8-40.9) | 38.1 (32.9-41.2) | 37.1 (31.675-40.125) | 0.174 |
| Hemoglobin |  | 12.6 (10.8-13.7) | 12.7 (11-13.8) | 12.15 (10.575-13.5) | 0.120 |
| Rdw |  | 13.8 (13.2-15) | 13.7 (13-14.9) | 14.05 (13.675-15.175) | 0.132 |
| RBC |  | 4.17 (3.6-4.56) | 4.19 (3.66-4.64) | 4.045 (3.448-4.36) | 0.092 |
| WBC |  | 10 (7.8-13) | 9.7 (7.7-12.8) | 11.35 (9.325-13.525) | 0.035 |
| Anion gap |  | 14 (12-16) | 14 (12-16) | 14 (12-16) | 0.656 |
| Calcium total |  | 8.9 (8.5-9.3) | 9 (8.5-9.3) | 8.5 (8.2-9.125) | 0.002 |
| Chloride |  | 104 (101-107) | 103 (100-107) | 104 (103-109) | 0.014 |
| Glucose |  | 129 (106-159) | 125 (106-154) | 151.5 (124.75-181.25) | 0.010 |
| Potassium |  | 3.9 (3.6-4.3) | 3.9 (3.7-4.3) | 4 (3.6-4.425) | 0.522 |
| Sodium |  | 139 (137-142) | 139 (137-142) | 140 (137.75-142.25) | 0.010 |
| Inrpt |  | 1.1 (1.1-1.2) | 1.1 (1.1-1.2) | 1.2 (1.1-1.325) | 0.019 |
| Pt |  | 12.4 (11.7-13.8) | 12.3 (11.6-13.4) | 13.2 (11.975-14.6) | 0.011 |
| Ptt |  | 27.7 (25.4-30.2) | 27.9 (25.6-30.1) | 26.8 (24.55-30.8) | 0.747 |
| ALT |  | 20 (14-34) | 20 (13-32) | 22.5 (15.5-50.5) | 0.001 |
| AST |  | 27 (19-40) | 25 (18-36) | 34 (22-61) | 0.003 |
| TBIL |  | 0.6 (0.4-0.9) | 0.6 (0.4-0.8) | 0.7 (0.5-1.125) | 0.020 |
| Creatinine |  | 0.9 (0.7-1.2) | 0.9 (0.7-1.1) | 0.9 (0.8-1.325) | 0.860 |
| Urea nitrogen |  | 16 (12-23) | 16 (12-22) | 18.5 (14.75-26) | 0.298 |
| Age |  | 70 (56-81) | 69 (56-80) | 74 (59.75-81.75) | 0.104 |
| Weight |  | 77.6 (64.3-95.1) | 78 (64.3-96.6) | 75.5 (64.275-86.75) | 0.444 |
| Sofa |  | 3 (1-4) | 2 (1-4) | 4 (2-6) | <0.001 |
| Apsiii |  | 33 (26-46) | 33 (26-45) | 36.5 (29.75-53.25) | <0.001 |
| Sapsii |  | 32 (25-38) | 31 (24-36) | 37 (31-44.25) | <0.001 |
| Gcs |  | 14 (12-15) | 14 (12-15) | 15 (11.75-15) | 0.779 |
| HR |  | 81 (72-92) | 81 (71-92) | 82.5 (73-92.25) | 0.723 |
| NBPS |  | 135 (121-150) | 136 (122-151) | 130 (111-140.5) | 0.029 |
| NBPD |  | 76 (63-87) | 76 (64-89) | 71.5 (61.75-83) | 0.223 |
| RR |  | 18 (15-22) | 18 (15-22) | 19 (15-22) | 0.804 |
| Spo2 |  | 98 (96-100) | 98 (96-100) | 99 (96-100) | 0.971 |
| Temperature |  | 98.3 (97.8-98.8) | 98.3 (97.8-98.7) | 98.35 (97.675-98.975) | 0.173 |
| Platelet count CV |  | 0.128 (0.063–0.209) | 0.113 (0.055–0.207) | 0.264 (0.077–0.387) | <0.001 |
| Platelet count |  | 219 (168-263) | 219.5 (173.5-264) | 214 (135.125-254) | 0.200 |
| ICH Location (%) | Deep hemorrhage | 20 (7.12) | 18 (7.86) | 2 (3.85) | 0.443 |
| ICH Location (%) | Cerebral lobe | 45 (16.01) | 37 (16.16) | 8 (15.38) |  |
| ICH Location (%) | Brainstem | 3 (1.07) | 2 (0.87) | 1 (1.92) |  |
| ICH Location (%) | Cerebellum | 13 (4.63) | 12 (5.24) | 1 (1.92) |  |
| ICH Location (%) | Ventricle | 36 (12.81) | 26 (11.35) | 10 (19.23) |  |
| ICH Location (%) | Multifocal hemorrhage | 2 (0.71) | 1 (0.44) | 1 (1.92) |  |
| ICH Location (%) | Others | 162 (57.65) | 133 (58.08) | 29 (55.77) |  |
| Gender (%) | Female | 125 (44.48) | 101 (44.10) | 24 (46.15) | 0.909 |
| Gender (%) | Male | 156 (55.52) | 128 (55.90) | 28 (53.85) |  |
| Race (%) | White | 3 (1.07) | 3 (1.31) | 0 (0.00) | 0.910 |
| Race (%) | Black | 2 (0.71) | 2 (0.87) | 0 (0.00) |  |
| Race (%) | Asian | 259 (92.17) | 209 (91.27) | 50 (96.15) |  |
| Race (%) | Other | 17 (6.05) | 15 (6.55) | 2 (3.85) |  |
| Htn (%) | No | 110 (39.15) | 88 (38.43) | 22 (42.31) | 0.719 |
| htn (%) | Yes | 171 (60.85) | 141 (61.57) | 30 (57.69) |  |
| Aki (%) | No | 235 (83.63) | 194 (84.72) | 41 (78.85) | 0.409 |
| aki (%) | Yes | 46 (16.37) | 35 (15.28) | 11 (21.15) |  |
| lc (%) | No | 273 (97.15) | 223 (97.38) | 50 (96.15) | 0.986 |
| lc (%) | Yes | 8 (2.85) | 6 (2.62) | 2 (3.85) |  |
| hep (%) | No | 277 (98.58) | 225 (98.25) | 52 (100.00) | 0.755 |
| Hep (%) | Yes | 4 (1.42) | 4 (1.75) | 0 (0.00) |  |
| pna (%) | No | 227 (80.78) | 190 (82.97) | 37 (71.15) | 0.079 |
| pna (%) | Yes | 54 (19.22) | 39 (17.03) | 15 (28.85) |  |
| ckd (%) | No | 240 (85.41) | 196 (85.59) | 44 (84.62) | 1.000 |
| ckd (%) | Yes | 41 (14.59) | 33 (14.41) | 8 (15.38) |  |
| Ca (%) | No | 231 (82.21) | 188 (82.10) | 43 (82.69) | 1.000 |
| ca (%) | Yes | 50 (17.79) | 41 (17.90) | 9 (17.31) |  |
| t2dm (%) | No | 199 (70.82) | 165 (72.05) | 34 (65.38) | 0.432 |
| t2dm (%) | Yes | 82 (29.18) | 64 (27.95) | 18 (34.62) |  |
| hld (%) | No | 170 (60.50) | 138 (60.26) | 32 (61.54) | 0.990 |
| hld (%) | Yes | 111 (39.50) | 91 (39.74) | 20 (38.46) |  |
| Hf (%) | No | 240 (85.41) | 191 (83.41) | 49 (94.23) | 0.075 |
| Hf (%) | Yes | 41 (14.59) | 38 (16.59) | 3 (5.77) |  |
| Mi (%) | No | 274 (97.51) | 223 (97.38) | 51 (98.08) | 1.000 |
| mi (%) | Yes | 7 (2.49) | 6 (2.62) | 1 (1.92) |  |
| copd (%) | No | 262 (93.24) | 215 (93.89) | 47 (90.38) | 0.547 |
| copd (%) | Yes | 19 (6.76) | 14 (6.11) | 5 (9.62) |  |
| Anti-coa (%) | No | 265 (94.31) | 215 (93.89) | 50 (96.15) | 0.760 |
| Anti-coa (%) | Yes | 16 (5.69) | 14 (6.11) | 2 (3.85) |  |
| Anti-pla (%) | No | 265 (94.31) | 215 (93.89) | 50 (96.15) | 0.760 |
| Anti-pla (%) | Yes | 16 (5.69) | 14 (6.11) | 2 (3.85) |  |
| Surgery (%) | No | 259 (92.17) | 210 (91.70) | 49 (94.23) | 0.744 |
| Surgery (%) | Yes | 22 (7.83) | 19 (8.30) | 3 (5.77) |  |
| CRRT (%) | No | 276 (98.22) | 226 (98.69) | 50 (96.15) | 0.504 |
| CRRT(%) | Yes | 5 (1.78) | 3 (1.31) | 2 (3.85) |  |
| Ventilation (%) | No | 101 (35.94) | 91 (39.74) | 10 (19.23) | 0.009 |
| Ventilation (%) | Yes | 180 (64.06) | 138 (60.26) | 42 (80.77) |  |
| Pla-transfusion (%) | No | 273 (97.15) | 222 (96.94) | 51 (98.08) | 1.000 |
| Pla-transfusion (%) | Yes | 8 (2.85) | 7 (3.06) | 1 (1.92) |  |

**Note**: Continuous variables are presented as median (interquartile range) and categorical variables as n (%). Platelet count CV is displayed on the original unitless scale and was calculated as the sample standard deviation divided by the mean of observed platelet measurements. N=281, including 229 survivors and 52 non-survivors. GCS, Glasgow Coma Scale; SOFA, Sequential Organ Failure Assessment; APS III, Acute Physiology Score III; SAPS II, Simplified Acute Physiology Score II; AKI, acute kidney injury; CKD, chronic kidney disease; CRRT, continuous renal replacement therapy.

**Table S3. Univariable Cox regression for 28-day mortality in the external validation cohort**

| **Characteristics** | **Hazard Ratio(HR)** | **lower_95** | **upper_95** | **p-value** |
| --- | --- | --- | --- | --- |
| Age | 1.015(0.997-1.034) | 0.997 | 1.034 | 0.104 |
| AKI |  |  |  |  |
| No |  |  |  |  |
| Yes | 1.379(0.709-2.682) | 0.709 | 2.682 | 0.344 |
| Anti-coa |  |  |  |  |
| No |  |  |  |  |
| Yes | 0.646(0.157-2.653) | 0.157 | 2.653 | 0.544 |
| Anti-pla |  |  |  |  |
| No |  |  |  |  |
| Yes | 0.634 | 0.154 | 2.607 | 0.528 |
| Apsiii | 1.027 | 1.013 | 1.041 | <0.001 |
| ca |  |  |  |  |
| No |  |  |  |  |
| Yes | 0.985 | 0.48 | 2.02 | 0.967 |
| Ckd |  |  |  |  |
| No |  |  |  |  |
| Yes | 1.049 | 0.494 | 2.227 | 0.902 |
| Copd |  |  |  |  |
| No |  |  |  |  |
| Yes | 1.657 | 0.659 | 4.165 | 0.283 |
| CRRT |  |  |  |  |
| No |  |  |  |  |
| Yes | 2.162 | 0.526 | 8.889 | 0.285 |
| ALT | 1.001 | 1 | 1.002 | 0.004 |
| Anion gap | 1.026 | 0.944 | 1.114 | 0.547 |
| AST | 1.001 | 1 | 1.001 | 0.005 |
| TBIL | 1.13 | 1.057 | 1.208 | <0.001 |
| Calcium | 0.571 | 0.402 | 0.813 | 0.002 |
| Chloride | 1.065 | 1.016 | 1.116 | 0.009 |
| Creatinine | 1.011 | 0.907 | 1.128 | 0.842 |
| Glucose | 1.006 | 1.002 | 1.01 | 0.007 |
| Hematocrit | 0.971 | 0.926 | 1.017 | 0.207 |
| Hemoglobin | 0.905 | 0.793 | 1.034 | 0.143 |
| HR | 1.003 | 0.988 | 1.019 | 0.688 |
| Inrpt | 3.005 | 1.139 | 7.931 | 0.026 |
| NBPD | 0.991 | 0.977 | 1.006 | 0.244 |
| NBPS | 0.986 | 0.973 | 0.999 | 0.033 |
| Potassium | 1.182 | 0.734 | 1.904 | 0.492 |
| Pt | 1.117 | 1.022 | 1.22 | 0.015 |
| Ptt | 0.994 | 0.966 | 1.023 | 0.69 |
| RDW | 1.091 | 0.969 | 1.229 | 0.149 |
| RBC | 0.737 | 0.512 | 1.062 | 0.101 |
| RR | 1.005 | 0.96 | 1.053 | 0.827 |
| Sodium | 1.085 | 1.028 | 1.146 | 0.003 |
| Spo2 | 0.996 | 0.894 | 1.11 | 0.945 |
| Temperature | 1.213 | 0.931 | 1.582 | 0.153 |
| Urea nitrogen | 1.008 | 0.994 | 1.022 | 0.292 |
| WBC | 1.056 | 1.008 | 1.106 | 0.021 |
| GCS | 1.015 | 0.918 | 1.123 | 0.77 |
| Gender |  |  |  |  |
| Female |  |  |  |  |
| Male | 0.939 | 0.544 | 1.62 | 0.821 |
| HF |  |  |  |  |
| No |  |  |  |  |
| Yes | 0.329 | 0.103 | 1.056 | 0.062 |
| HLD |  |  |  |  |
| No |  |  |  |  |
| Yes | 0.97 | 0.555 | 1.696 | 0.915 |
| HTN |  |  |  |  |
| No |  |  |  |  |
| Yes | 0.865 | 0.499 | 1.499 | 0.604 |
| ICH Location |  |  |  |  |
| Deep hemorrhage |  |  |  |  |
| Cerebral lobe | 1.878 | 0.399 | 8.843 | 0.425 |
| Brainstem | 3.485 | 0.316 | 38.446 | 0.308 |
| Cerebellum | 0.746 | 0.068 | 8.226 | 0.811 |
| Ventricle | 3.095 | 0.678 | 14.125 | 0.145 |
| Multifocal hemorrhage | 7.52 | 0.681 | 83.065 | 0.1 |
| Others | 1.919 | 0.458 | 8.04 | 0.373 |
| Platelet count | 0.998 | 0.994 | 1.001 | 0.193 |
| lc |  |  |  |  |
| No |  |  |  |  |
| Yes | 1.424 | 0.346 | 5.852 | 0.624 |
| mi |  |  |  |  |
| No |  |  |  |  |
| Yes | 0.694 | 0.096 | 5.025 | 0.718 |
| Platelet count CV, per 0.1-unit increase | 1.984 (1.588–2.478) | 1.588 | 2.478 | <0.001 |
| Platelets transfusion |  |  |  |  |
| No |  |  |  |  |
| Yes | 0.658 | 0.091 | 4.764 | 0.679 |
| PNA |  |  |  |  |
| No |  |  |  |  |
| Yes | 1.685 | 0.925 | 3.071 | 0.088 |
| Sapsii | 1.049 | 1.031 | 1.068 | <0.001 |
| sofa | 1.199 | 1.106 | 1.3 | <0.001 |
| Surgery |  |  |  |  |
| No |  |  |  |  |
| Yes | 0.663 | 0.207 | 2.128 | 0.49 |
| T2DM |  |  |  |  |
| No |  |  |  |  |
| Yes | 1.301 | 0.735 | 2.303 | 0.367 |
| Ventilation |  |  |  |  |
| No |  |  |  |  |
| Yes | 2.432 | 1.22 | 4.849 | 0.012 |
| Weight | 0.993 | 0.981 | 1.006 | 0.305 |

**Note**: The hazard ratio for platelet count CV is reported per 0.1-unit increase. GCS, Glasgow Coma Scale; SOFA, Sequential Organ Failure Assessment; APS III, Acute Physiology Score III; SAPS II, Simplified Acute Physiology Score II; AKI, acute kidney injury; CKD, chronic kidney disease; CRRT, continuous renal replacement therapy; CI, confidence interval; HR, hazard ratio.

**Table S4. ICU-period landmark sensitivity analyses for platelet count variability and mortality**

| **Analysis** | **Outcome** | **Included, N** | **Excluded, N** | **HR (95%CI)** | **P-value** |
| --- | --- | --- | --- | --- | --- |
| 3-day landmark Cox model | 28-day mortality | 2088 | 114 | 1.42 (1.25-1.61) | < 0.001 |
| 3-day landmark Cox model | 90-day mortality | 2088 | 114 | 1.34 (1.17-1.56) | < 0.001 |
| 3-day landmark Cox model | 360-day mortality | 2088 | 114 | 1.27 (1.12-1.44) | < 0.001 |
| 7-day landmark Cox model | 28-day mortality | 1993 | 209 | 1.39 (1.14–1.69) | < 0.001 |
| 7-day landmark Cox model | 90-day mortality | 1993 | 209 | 1.27 (1.11-1.45) | < 0.001 |
| 7-day landmark Cox model | 360-day mortality | 1993 | 209 | 1.14 (1.02-1.29) | 0.024 |

**Note**: Platelet count CV was recalculated using observed platelet measurements obtained within the first 3 or 7 days after ICU admission. Patients who died before the landmark or had fewer than three measurements within the corresponding window were excluded. Hazard ratios are reported per 0.1-unit increase in platelet count CV.

**Table S5. Imaging-adjusted sensitivity analysis in the external validation cohort**

**Table S5 (A) Imaging characteristics according to 28-day vital status**

| **Variable** | **Non-death within 28 days N=229** | **Death within 28 days N=52** | **P-value** |
| --- | --- | --- | --- |
| Hematoma volume, mL | 30.21 (6.24-47.21) | 47.32 (28.90-63.54) | <0.001 |

**Table S5 (B) Imaging-adjusted Cox models**

| **Outcome** | **Model** | **Adjusted HR for platelet count CV** | **95% CI** | **P-value** |
| --- | --- | --- | --- | --- |
| 28-day mortality | Multivariable Cox model | 1.37 | 1.12–1.59 | <0.001 |
| 90-day mortality | Multivariable Cox model | 1.26 | 1.07–1.49 | 0.006 |
| 360-day mortality | Multivariable Cox model | 1.17 | 1.02–1.32 | 0.017 |

Note: All 281 patients in the external validation cohort had complete imaging data from admission CT examinations; 229 were alive and 52 had died within 28 days. Hematoma volume was estimated from the admission CT reports interpreted by experienced senior clinical radiologists. Cox models were adjusted for age, sex, GCS, SOFA, baseline platelet count, platelet measurement count, acute kidney injury, pneumonia, chronic kidney disease, myocardial infarction, anticoagulant use, surgery, CRRT, mechanical ventilation, platelet transfusion, ICH location, race, hematoma volume, and intraventricular hemorrhage. Hazard ratios are reported per 0.1-unit increase in platelet count CV. Residual confounding by hematoma expansion, formal ICH score, withdrawal-of-care decisions, and other incompletely measured neurological prognostic factors cannot be excluded.

**Table S6. Comparison of included patients and excluded patients with one or two eligible platelet measurements**

| **Variable** | **Included (≥3 measurements)** | **Excluded (1–2 measurements)** | **SMD** |
| --- | --- | --- | --- |
| Age, years | 70.00 (58.00–80.00) | 73.00 (60.00–83.00) | -0.146 |
| Weight, kg | 76.20 (64.22–90.29) | 75.00 (62.20–87.43) | 0.021 |
| GCS | 14.00 (11.00–15.00) | 15.00 (12.00–15.00) | 0.010 |
| SOFA | 3.00 (2.00–5.00) | 3.00 (1.00–5.00) | -0.042 |
| APS III | 35.00 (26.00–45.00) | 36.00 (26.00–50.00) | -0.153 |
| SAPS II | 32.00 (25.25–40.00) | 34.00 (26.00–43.00) | -0.237 |
| Baseline platelet count, K/μL | 209.00 (165.00–259.00) | 205.00 (163.00–254.00) | 0.036 |
| Platelet measurement count | 8.00 (5.00–13.00) | 2.00 (1.00–2.00) | 2.342 |
| Male | 1195 (54.3%) | 253 (48.9%) | 0.107 |
| Platelet transfusion | 119 (5.4%) | 21 (4.1%) | 0.063 |
| AKI | 439 (19.9%) | 37 (7.2%) | 0.380 |
| Pneumonia | 513 (23.3%) | 29 (5.6%) | 0.520 |
| Surgery | 196 (8.9%) | 12 (2.3%) | 0.289 |
| CRRT | 48 (2.2%) | 2 (0.4%) | 0.160 |
| Mechanical ventilation | 1545 (70.2%) | 255 (49.3%) | 0.435 |
| In-hospital mortality | 294 (13.4%) | 222 (42.9%) | -0.697 |
| 28-day mortality | 284 (12.9%) | 227 (43.9%) | -0.732 |
| Race |  |  |  |
| White | 1316 (59.8%) | 322 (62.3%) | -0.052 |
| Black | 220 (10.0%) | 42 (8.1%) | 0.065 |
| Asian | 90 (4.1%) | 17 (3.3%) | 0.042 |
| Others | 576 (26.2%) | 136 (26.3%) | -0.003 |
| ICH location |  |  |  |
| Deep hemorrhage | 169 (7.7%) | 34 (6.6%) | 0.043 |
| Cerebral lobe | 386 (17.5%) | 89 (17.2%) | 0.008 |
| Brainstem | 43 (2.0%) | 16 (3.1%) | -0.073 |
| Cerebellum | 110 (5.0%) | 24 (4.6%) | 0.016 |
| Ventricle | 257 (11.7%) | 45 (8.7%) | 0.098 |
| Multifocal hemorrhage | 21 (1.0%) | 4 (0.8%) | 0.019 |
| Others | 1216 (55.2%) | 305 (59.0%) | -0.076 |

**Note:** Included N=2,202. Among the 761 patients excluded for having fewer than three eligible pre-ICU platelet measurements, patient-level covariates were available for 517 patients with one or two measurements and were unavailable for the remaining 244 patients. An absolute value of the standardized mean difference (SMD) ≥0.10 was considered potentially meaningful.

**Table S7. Fixed-three-measurement sensitivity analysis for mortality outcomes**

| **Outcome** | **N** | **Events** | **Effect measure** | **Fixed last-three CV: clinically adjusted model (95% CI)** | **P-value** | **Fixed last-three CV: clinically + monitoring-adjusted model (95% CI)** | **P-value** |
| --- | --- | --- | --- | --- | --- | --- | --- |
| In-hospital mortality | 2202 | 294 | OR per 0.1 CV | 1.33 (1.15–1.53) | <0.001 | 1.26 (1.09–1.46) | 0.002 |
| 28-day mortality | 2202 | 284 | HR per 0.1 CV | 1.25 (1.14–1.38) | <0.001 | 1.22 (1.11–1.36) | <0.001 |
| 90-day mortality | 2202 | 326 | HR per 0.1 CV | 1.20 (1.09–1.32) | <0.001 | 1.17 (1.06–1.29) | 0.001 |
| 360-day mortality | 2202 | 347 | HR per 0.1 CV | 1.18 (1.07–1.29) | <0.001 | 1.15 (1.05–1.27) | 0.004 |

*Note: Fixed-three CV was calculated from the last three nonmissing pre-ICU platelet measurements for each patient. The clinically adjusted model included age, sex, GCS, SOFA, baseline platelet count, acute kidney injury, pneumonia, chronic kidney disease, myocardial infarction, anticoagulant use, surgery, CRRT, mechanical ventilation, platelet transfusion, ICH location, and race. The monitoring-adjusted model additionally included the total number of eligible pre-ICU platelet measurements. ORs and HRs are reported per 0.1-unit increase in platelet count CV.*

**Figure S1. Monitoring-adjusted multivariable Cox proportional hazards models for (A) 28-day, (B) 90-day, and (C) 360-day mortality. Hazard ratios for platelet count CV are reported per 0.1-unit increase. All models included platelet measurement count. CI, confidence interval; CV, coefficient of variation; HR, hazard ratio.**
